# Analysis of the Influencing Factors on the Preferences of the Elderly for the Combination of Medical Care and Pension in Long-Term Care Facilities Based on the Andersen Model

**DOI:** 10.3390/ijerph17155436

**Published:** 2020-07-28

**Authors:** Yong Wei, Liangwen Zhang

**Affiliations:** 1 Department of Statistics, School of Economics, Xiamen University, 422 Siming South Road, Xiamen 361005, China; weiyong0816@163.com; 2 School of Accounting, Jiangsu Vocational College of Finance and Economics, Huaian 223003, China; 3 State Key Laboratory of Molecular Vaccinology and Molecular Diagnostics, School of Public Health, Xiamen University, Xiamen 361102, China

**Keywords:** combination of medical care and pension, care needs, influencing factors, Andersen model, elderly

## Abstract

Background: The purpose of this study is to evaluate the status quo and factors that influence the preferences of the elderly for the combination of medical care and pension (CMCP) in long-term care (LTC) facilities and to provide an evidence-based basis for building a multi-tiered, continuous LTC system with CMCP. Methods: Using a multi-stage sampling method, face-to-face questionnaire surveys were conducted on 3260 elderly people aged 60 years or over in 44 communities in 16 sub-districts in six districts in Xiamen. Based on the Andersen model, the chi-square test was used to analyze differences in population distribution, and binary logistic regression analysis was used to analyze the factors affecting the elderly’s preference for CMCP in LTC institutions in terms of the factors of predisposition, enablement, and personal needs. Results: Most elderly people choose traditional home care (82.01%), and only 12.89% choose LTC facilities with CMCP. This choice is influenced by a number of predisposing factors. The elderly who are at the upper end of the age range, have a higher education level, and live in rural areas are more likely to choose CMCP (odds ratio (OR) value greater than 1, *p* < 0.05). With regard to enabling factors, the elderly who were married, mainly taken care of by spouses, and had better economic status also tended to choose CMCP (OR > 1, *p* < 0.01). In terms of personal needs, the elderly with worse self-care status tended to choose CMCP (OR > 1, *p* < 0.01). Enabling factors have the largest contribution to the model, and they have the greatest impact on elder preference for CMCP services. In addition, the elderly with higher age and education level, non-remarried, with better economic status, and with poorer health status have a demand for a wider variety of CMCP services. Compared to those in urban areas, the elderly in rural areas have greater needs, mainly related to personal care, medical care, and psychological counseling. Conclusion: The preference of the elderly for CMCP are lower compared to their preference for home care in Xiamen, China. Preference for CMCP is affected by a range of factors such as age, education level, residence, income, and self-care ability, among which the enabling factors have the greatest impact.

## 1. Introduction

The aging of China’s population has become an increasingly serious problem. The number of incapacitated or semi-incapacitated elderly people is steadily rising, increasing the demand for old-age care and medical services. By the end of 2016, China’s elderly population aged 60 and above reached 240 million, accounting for 17.3% of the total population [1]. Among these, more than 40 million are incapacitated or semi-incapacitated, accounting for 18.3% of the total elderly population [2]. At present, China has entered a rapid development period of population aging. According to United Nations statistics, in 2015 it took roughly 25 years for the proportion of the Chinese population over 65 years old to increase from 7% to 14%, a rate which is much faster than that found in developed countries (France, 115 years; Sweden, 85 years; USA, 69 years; UK, 45 years) [3]. It is clear, then, that China’s rapid ageing poses a host of challenges. 

The aging population has led to a surge in demand for care for the elderly. With the continuous extension of life expectancy, physical and mental health problems have become increasingly prominent. The average life expectancy in China in 2018 was 77 years, while the average healthy life expectancy was only 68.7 years, a gap of about 10 years [4]. This shows that the health status of the elderly is poor, and physical and mental health problems are prominent issues for them. In addition, China has a high rate of disability. It is predicted that the total disabled population will increase rapidly, rising from 43.75 million in 2020 to 91.4 million in 2050. Of that total, 69.7% are expected to be urban elderly. By 2050, the population of elderly individuals with mild, moderate, or severe disabilities will be 108%, 104%, and 120%, respectively, as compared to their 2020 totals [5]. The disability rate of the elderly in China is thus expected to increase rapidly, with a concomitant deepening of the severity of disability. This combination of an increase in life expectancy, an aging population, a high prevalence of chronic diseases in old age, a serious problem of disability, and low healthy life expectancy has led to a surge in the demand for care for the elderly.

Numerous previous studies have shown that China’s elderly have the greatest demand for life care and medical care compared to other care services, such as spiritual consolation, social activities, etc. [6,7]. Since 2016, the Chinese government has promoted the important development strategy of “the Combination of Medical Care and Pension” (CMCP) as a way to cope with the rapid aging of the society, as announced in the *Healthy China 2030 Plan* and the *13th Five-year Development Plan*. It focuses on promoting a combination of medical care and nursing care, healthy aging, and vigorous development of services and industries for the elderly [8]. For the long-term care (LTC) model, it mainly emphasizes the carrier of service, such as home care, community care and institutional care. As a new type of LTC service, CMCP focuses on the integration of different care services. CMCP is coping with one of the important means to the healthy development of the aging society and is after readjustment endowment services, unlike the traditional home care model in China, to integrate optimization, medical care and pension resources for the elderly, which includes providing daily care, nursing care, medical services, and other forms from the new LTC service model in order to achieve healthy aging [9].

There have been many studies on the need for elderly care services, but few studies have examined the need for CMCP in long-term care (LTC) facilities. The elderly in China tend to prefer to live at home, a setting that has been the focus of most prior studies. Family pensions are favored by the vast majority of the elderly, much more so than other pension modes [10,11,12]. At the same time, the existing research on CMCP also focuses on its concept elaboration, mode selection, policy analysis [13,14]. The understanding and practice of CMCP in China are mainly limited to LTC or medical institutions, with a focus on the elderly with poor health or disabilities. However, elderly people at different life stages have different demands for CMCP, and CMCP services for the elderly should run through the entirety of old age [15,16]. In addition, there are few studies on the factors which influence demand for CMCP [17,18], differences in the needs for specific CMCP services among the elderly, as well as a lack of any relevant framework for theoretical analysis. An analysis of how different variables affect the needs of the elderly for CMCP services can provide a reference for policymakers to optimize resource allocation and to introduce targeted, scientific, and sustainable pension policies. 

Therefore, this study aims to explore the influence of different factors on the preferences of the elderly for CMCP endowment services, and to better understand what services they need. It will help to give full play to the important supplementary role of institutional care, thereby improving China’s multi-level pension system, including families, communities and institutions. At last, this paper aims to provide a reference to enable policymakers to optimize resource arrangement and ensure the sustainability of China’s old-age security policy.

## 2. Materials and Methods

### 2.1. Study Design

In May 2016, the total population of Xiamen reached 2.2055 million, of which the elderly population over 60 years old accounted for 14%. In July–October 2016, the project team surveyed the elderly aged 60 and over in Xiamen City (residents of more than 6 months), using a multi-stage stratified sampling method to select 44 communities in 16 sub-districts in 6 districts of Xiamen City. After receiving identical training, investigators conducted a questionnaire survey on the elderly in the selected communities through face-to-face interviews. Questions mainly included the elderly’s sociodemographic variables, family conditions, health status, and CMCP service preferences. The inclusion criteria of the subjects in this study were as follows: (1) home-based elderly; (2) age ≥ 60 years; (3) able to communicate effectively; (4) gave informed consent. Exclusion criteria: (1) inability to communicate effectively; (2) living in LTC institutions. 

The quality control strategy adopted in this survey was as follows: (1) During the research design phase, expert consultation and discussion ensured the scientific legitimacy and feasibility of the research plan and questionnaire. (2) Pre-surveys were conducted to improve the questionnaire, to develop standard operating procedures, and to develop a consistent training protocol for investigators. (3) During on-site investigations, quality control personnel were set up to conduct on-site audits and quality control, and to adopt double-blind protocols for later data entry and verification.

### 2.2. Andersen Theoretical Model 

**Figure 1:**
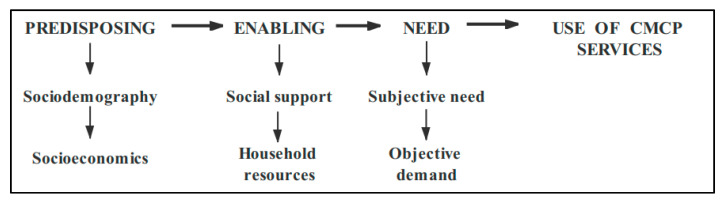
The Andersen theoretical model for influencing factors of the combination of medical care and pension (CMCP) service needs.

Based on Andersen’s model of healthy behavior [19], this study explores a theoretical framework for the analysis of factors that influence CMCP service preferences among the elderly (Figure 1). This model is widely used in studies of healthcare demand. The factors of influence were subdivided into factors that predispose elderly individuals to choose CMCP, factors that enable them to successfully make use of CMCP, and factors of personal needs that influence demand for CMCP. On the basis of the Andersen behavior model, this paper constructed the adjusted Andersen theoretical model of influencing factors on CMCP needs according to the research purpose and variable information availability: (1) predisposing factors affecting model variables, including type of residence, gender, age, and level of education; (2) enabling variables, including living style, marriage, number of children, income, primary caregivers, and type of medical insurance; (3) personal needs variables, including self-assessment of health, activities of daily living (ADL), and feelings of loneliness.

#### 2.2.1. Dependent Variables

CMCP preferences were the central dependent variable, consisting of two dimensions: (1) anticipated need for CMCP and (2) anticipated need for specific CMCP services. The question measuring the preference for CMCP was phrased as: “Do you want to live in an LTC facility with CMCP?” The anticipated need for specific CMCP services was measured using the question: “If you live in a CMCP facility, what kind of service would you like to receive?” The answer options included (1) personal care services, (2) medical care, (3) health examination, (4) psychological consulting, (5) social and recreational activities, and (6) health education.

#### 2.2.2. Independent Variables

This paper constructed an adjusted Andersen theoretical model of influencing factors on CMCP preference according to the purpose of this research and the availability of information regarding the relevant variables. The predisposing variables were age, gender, residence (urban, town, or rural), current living arrangement (whether or not living alone), and educational level (years of schooling). The enabling variables were expressed by occupation (farmer vs. others), marital status (married vs. unmarried), economic status (income exceeded expenditures, balanced, expenditures exceeded income), primary caregivers (spouse, children, others, none), number of children (0, 1, 2 or more), and medical insurance (yes vs. no). Personal needs variables included activities of daily living (not limited, limited, strongly limited), feelings of loneliness (often, sometimes, seldom/never), and self-rated health status (good vs. bad).

### 2.3. Analytical Methods

Descriptive statistics were presented for distribution characteristics of the surveyed subjects and the distribution of CMCP preferences. The chi-square test was then used to analyze differences in the distribution of different CMCP preferences of the elderly. Taking the variables with chi-square test *p* < 0.1 as the independent variables, a binary logistic regression model was constructed to analyze the factors that influence the elderly’s preferences for CMCP services. First, the predisposing variables were taken as control variables and put into model 1 as the benchmark model. Then, based on the benchmark model, enabling variables and personal needs variables were added successively to form model 2 and model 3, to explain the variation of the variance of the dependent variables by the addition of other types of factors to the benchmark model. Finally, model 4 was constructed with all variables as independent variables to determine the overall explanatory power of the model. In addition, 4 binary logistic regression models (Model I–IV) were constructed by including all three types of independent variables (predisposing, enabling, and personal needs) into the regression model and eliminating the independent variables one by one, so as to compare the relative influence of the three kinds of factors. The specific models are as follows:

Model I: *Logit* (*Yi*) = predisposing variables + enabling variables + need variables

Model II: *Logit* (*Yi*) = enabling variables + need variables

Model III: *Logit* (*Yi*) = predisposing variables + need variables

Model IV: *Logit* (*Yi*) = predisposing variables + enabling variables

All statistical analyses were conducted using SAS 9.3. The odds ratio and 95% confidence interval indicated the effect of each predictor and whether it met statistical significance. A value of *p* < 0.05 was considered statistically significant.

## 3. Results

### 3.1. Predisposing, Enabling, and Personal Needs Variables

A total of 3160 questionnaires were completed of which 3119 were valid, giving an effective rate of 98.7%. Among these, males accounted for 51.0%. The mean age was 70.27 ± 7.81 years old. A total of 35.8% of the elderly live in rural areas, 40.1% cannot read, 30.2% are unmarried, 1.0% have no children, 16.6% have daily expenses that exceed their income, 29.8% have no caregivers, 5.7% are incapacitated, and 39.2% reported often feeling lonely.

### 3.2. Status of CMCP

Among the 3119 elderly respondents, 402 (12.89%) indicated that they wanted to be admitted to a CMCP nursing facility, 82.01% preferred home-based care, and 5.1% preferred community care. The analysis of the distribution of elderly people’s preference for different life care options shows that differences in preference depend on factors such as residence, age, education level, occupation, number of existing children, marital status, income and expenditure, primary caregivers, and activities of daily life (Table 1).

### 3.3. Factors Influencing CMCP Service Needs

Table 2 shows the binary logistic regression analysis of factors influencing CMCP service preferences. In the predisposing factors, differences in residence, age, and education level were found to be statistically significant. It was found that the elderly living in rural areas have a higher preference for CMCP services than those in urban areas, (odds ratio (OR) = 2.1, 2.07, 2.3, 2.16) and that the OR values are greater than those in the town. As compared to the 60–69 years age range, the elderly in the 70–79 age group have a higher demand for CMCP services (OR = 2.21, 1.65, 1.74), but the difference between these two groups is not statistically significant. With regard to literacy, the CMCP service preference of those with only an elementary school education is lower (OR value is less than 1,) as compared to those above the high school level (OR value is greater than 1.)

Marriage, income and expenditure, and primary caregivers are among the enabling factors that proved to be statistically significant in Models 2 and 4. The OR values of the CMCP service needs of unmarried elderly are 1.61 and 1.64, respectively. The more an individual’s income exceeded their expenditures, the greater the OR value of their preference for CMCP service. For the elderly who are mainly cared for by their spouses, the OR value of preference for CMCP service is less than 1, while the elderly who are cared for by others (such as son-in-law or nephew) are more willing to accept CMCP service (OR values of 2.05 and 1.77, respectively.) Activities of daily life was a statistically significant factor in Models 3 and 4. As compared to the elderly who were completely independent, the worse the degree of capacity for self-care, the greater was the OR value of preference for CMCP.

Table 3 shows the comparison of prediction probabilities and goodness of fit of each model. Compared with Model I, the changes of -2LL (-2 Log Likelihood) in Models II and III are the largest, and the changes of Cox and Snell R2 and Nagelkerke R2 in Models III are similar. These indicate that the enabling factors contribute the most to Model I among the three types of influencing factors; the second-largest contributors were the predisposing factors.

The results of this study show that, in general, the elderly have higher needs for life care and medical care than others (Table 4). The elderly with higher age and education level, non-remarried, with better economic status, and with poorer health status have a demand for a wider variety of CMCP services. Compared to those in urban areas, the elderly in rural areas have greater needs, mainly related to personal care, medical care, and psychological counseling. In addition, the elderly with moderate to severe disabilities tend to choose medical care services such as medical care, health examination, and health education.

## 4. Discussion

### 4.1. CMCP Needs of the Elderly

In this survey, the majority of the elderly still chose traditional home-based care (82.01%), consistent with the results of previous studies [20,21,22]. One can infer that, under the influence of traditional Chinese ideology and culture (such as filial piety) and long historical practice, family care will remain the most important way of providing for the aged for a considerable time. The government should, therefore, focus on strengthening policy support for family care. Moreover, socialized pension modes such as CMCP emerged in the process of industrialization in western developed countries and has a relatively short history of development in China. As a result, it is a comparatively new pension mode unfamiliar to the elderly [23,24].

However, it is worth noting that in this survey, the proportion of elderly people choosing CMCP institutions is much higher than the proportion of elderly people willing to choose CMCP institutions, as surveyed by our project team in 2013 (2.86%) [25,26,27]. A cross-sectional survey was performed by our project team across 173 communities in Xiamen, China, in 2013. A total of 261,043 individuals were aged ≥ 60 years in Xiamen at the survey time and about 5.5% of overall elderly populations were covered in that survey. In total, 14,292 valid questionnaires were obtained. The results of the two surveys reflect the fact that, with the vigorous advocacy and policy promotion of CMCP service by the Chinese government, the elderly have gradually realized the advantages of institutional care. It can solve a range of elder care needs caused by rapid aging of the population and by family miniaturization, taking into account both the “medical” and “care” aspects of the elderly’s most serious concerns [28]. Therefore, the government should formulate and improve the relevant CMCP policies as soon as possible, establish a coordination department for medical care, improve access and service standards, explore innovative service models, improve the service quality of pension institutions, and enhance the public’s awareness and acceptance of the CMCP model.

### 4.2. Factors Influencing Preference for CMCP Service

Most of the existing research on CMCP preference lacks the support of theoretical models and does not classify impact factors. Individual studies have only classified the factors simply, and did not conduct comparative studies on different categories of factors [29,30,31]. Based on the Andersen model, this paper constructed a framework for theoretical analysis of the elderly’s preferences for CMCP services. Through field investigation of the elderly in Xiamen community in China, this study measured the relative influence of factors of predisposition, enabling, and personal need on the elderly’s elder care choices. As a class, enabling factors have the greatest influence, followed by predisposing factors, indicating that personal family resources are the main factor affecting CMCP service choices.

This study found that older people with higher age and education are more willing to accept CMCP services. With the increase in age, the elderly’s sense of loneliness becomes stronger, their sense of security gradually weakens, and their ability to take care of themselves gradually declines [32,33]. With increase in education, the elderly become more aware of and more accepting of CMCP institutional care, whereas the less educated tend to have more conservative ideas. In contrast to previous research [34,35], this study found higher acceptance of the CMCP endowment institutions in rural populations. The main reason for this may be that the phenomenon of rural empty-nest in the process of rapid urbanization is serious, and there is a lack of existing old-age resources in rural areas. This makes it difficult to satisfy the life care and medical care needs of most of the elderly in those areas.

The results of this study show that elderly people who are poor and are mainly cared for by spouses have a lower willingness to choose institutional care services, while those elderly who are better off or who are mainly cared for by others (other than spouses) are more willing to accept CMCP institutional care services. The reason may be that the spouse is the person most familiar with the elderly individual, and the choice of life care can be more flexible. The married elderly people can meet their most important life care and spiritual comfort needs with the help of their spouse [36,37]. Due to the influence of traditional Chinese ideas, they are more willing to accept informal care services from their families. In addition, economic ability is a factor that attracts substantial attention in the relevant studies on pension choice. Most of the social pension services are currently paid services, which means that economic level is a limiting factor for the elderly who choose the social pension. Many studies [38,39] have shown that the elderly with higher economic status are more likely to choose the social pension because of their strong ability to pay for pension services, and this is further confirmed by this study.

Self-care ability largely determines the medical service needs of the elderly: the worse their capability for self-care, the greater their medical service needs. Losing the capacity for self-care is almost an inevitable life course for everyone. As a social public service to improve the quality of life of the elderly, CMCP service has been gradually accepted by the public [40,41]. CMCP endowed institutions are able to provide the elderly with more professional care services and with medical resources as well. This is particularly important for the elderly in poor health, and is an important reason to promote the choice of social endowment. Therefore, this study found that older people with poor self-care ability were more willing to choose CMCP institutions for the elderly. A unified long-term care insurance system urgently needs to be established by Chinese policymakers to reduce the financial burden of the elderly.

Consistent with previous results in other countries [42,43], this study found that the elderly generally have a higher demand for life care and medical care than in the past, thus indicating the importance of China’s efforts to promote the CMCP development strategy. Among them, the elderly with higher age and education level, who are non-remarried, and have better economic status and worse health condition have a higher demand for a range of CMCP services. Compared to the urban elderly, the rural elderly people have more serious needs, which are mainly reflected in daily care, medical care, and psychological counseling. In addition, moderately and severely disabled elderly people tend to choose medical care, health examination, and health education, among other medical care services. These results suggest that we should focus on elderly people in rural areas who are living in empty nests or alone and in poor health, and meeting their service needs such as daily care, medical care, and spiritual comfort.

However, there were several limitations to this study. First, the data were a cross-sectional survey, and this limited the interpretation of the results, making it hard to draw causal conclusions. Second, the question measuring the preference for CMCP and the exclusion criteria for the elderly under investigation (inability to communicate effectively) may have a selective bias in this study. Third, the models explain a low proportion of the variance, suggesting that other factors aside from those included in our analysis influence the various aspects of CMCP needs. Fourth, the participants were sampled from a single city in China. The results may reflect specific characteristics of that city, or at the very least introduce a rural–urban bias into the interpretation of the data. Therefore, longitudinal studies should be conducted to assess policy trends and key stakeholders.

## 5. Conclusions

In summary, the preference of the elderly for CMCP is lower compared to their preference for home care in Xiamen, China. Preference for CMCP is affected by a range of factors such as age, education level, residence, income, and self-care ability, among which the enabling factors have the greatest impact. Given the large-scale trends of an aging population and the weakening of the family’s traditional elder care function, the elderly’s demand for CMCP services is increasing gradually, particularly with regard to life care, medical care, mental comfort, and health intervention. The CMCP old-age care model has become an important choice to solve the problems of caring for an aging population. However, in the process of providing CMCP old-age care services, it is necessary to proceed from an understanding of the different needs of different elderly demographics. In China, although the function of family care has gradually weakened, it has a profound historical foundation. At the present stage, the government should be committed to establishing a multi-level CMCP service model based on family care, relying on community care, and supplemented by institutional care, with the focus on building family care service networks and improving the relevant supporting measures. In the face of the current differences in China’s urban and rural economic levels, uneven distribution of old-age and medical resources, and the accelerated urbanization process, there is a strong demand for elderly care services in rural areas, but the current status of the provision of elderly care services is worrying. Thus, China should continue to encourage society to implement CMCP institutional pension services with a focus on rural areas. It should also increase capital investment, policy support, and infrastructure construction for elder care. In the primary stage of carrying out CMCP elderly care service, policymakers should take the disabled and poor elderly as the key service subjects, actively provide policy care, provide multiple channels for elder care, and ensure the fulfillment of CMCP elder care needs, so as to improve the utilization rate of social elder care services. Finally, the government must strengthen the supervision of service quality, improve service standardization, comprehensively improve the quality of CMCP elderly care services, enhance their appeal to the elderly, and improve their willingness to socialize elder care, so as to promote the sustainable development of the elder care industry.

## Figures and Tables

**Table 1:** Different distribution characteristics of the elderly CMCP needs N(%).

Variables		CMCP Needs		Total	*χ* ^2^	*P*
		Yes (*n* = 402)	No (*n* = 2717)			
Residence	Urban	244 (60.7)	1327 (48.8)	1571 (50.3)	38.88	<0.001
(*n* = 3119)	Town	70 (17.4)	362 (13.3)	432 (13.9)		
	Rural	88 (21.9)	1028 (37.8)	1116 (35.8)		
Gender	Male	207 (51.5)	1382 (50.9)	1589 (51.0)	0.05	0.81
(*n* = 3119)	Female	195 (48.5)	1335 (49.1)	1530 (49.0)		
Age (year)	60~	280 (69.7)	1450 (53.4)	1730 (55.5)	40.20	<0.001
(*n* = 3119)	70~	90 (22.4)	825 (30.4)	915 (29.3)		
	80~	32 (8.0)	442 (16.3)	474 (15.2)		
Education	Illiterate	107 (27.0)	1134 (42.0)	1241 (40.1)	33.55	<0.001
(*n* = 3097)	Primary	132 (33.2)	736 (27.3)	868 (28.0)		
	Junior high school	85 (21.4)	473 (17.5)	558 (18.0)		
	Senior high school and above	73 (18.4)	357 (13.2)	430 (13.9)		
Living alone	No	345 (87.6)	2301 (88.6)	2646 (88.4)	0.34	0.56
(*n* = 2992)	Yes	49 (12.4)	297 (11.4)	346 (11.6)		
Occupation	Farmer	137 (34.1)	1349 (49.9)	1486 (47.8)		<0.001
	Non-farmer	265 (65.9)	1357 (50.1)	1622 (52.2)		
Number of children	0	6 (1.5)	26 (1.0)	32 (1.0)	40.92	<0.001
(*n* = 3107)	1	118 (29.5)	448 (16.5)	566 (18.2)		
	≥2	276 (69.0)	2233 (82.5)	2509 (80.8)		
Marital status	Married	294 (76.2)	1779 (68.8)	2073 (69.8)	8.65	0.003
(*n* = 2992)	Unmarried	92 (23.8)	807 (31.2)	899 (30.2)		
Economic status	Expenditures exceeded income	73 (18.2)	440 (16.3)	513 (16.6)	13.38	0.04
(*n* = 3096)	Balance	280 (69.7)	1882 (69.9)	2162 (69.8)		
	Income exceeded expenditures	49 (12.2)	372 (13.8)	421 (13.6)		
Primary caregiver	None	140 (40.1)	686 (28.3)	826 (29.8)	21.89	<0.001
(*n* = 2771)	Spouse	128 (36.7)	1003 (41.4)	1131 (40.8)		
	Children	78 (22.3)	685 (28.3)	763 (27.5)		
	Others	3 (0.9)	48 (2.0)	51 (1.9)		
Medical insurance	No	16 (4.1)	142 (5.4)	158 (5.2)	4.35	0.23
(*n* = 2992)	Yes	372 (95.9)	2478 (94.6)	2850 (94.8)		
Self-rated health	Bad	49 (12.3)	399 (14.8)	448 (14.5)	2.10	0.08
(*n* = 3088)	Good	349 (87.7)	2291 (85.2)	1232 (85.5)		
ADL	Completely independent	364 (96.0)	2164 (85.9)	2528 (87.2)	30.54	<0.001
(*n* = 2899)	Relatively independent	8 (2.1)	199 (7.9)	207 (7.1)		
	Disability	7 (1.8)	157 (6.2)	164 (5.7)		
Feeling of loneliness	Often	147 (36.6)	1077 (39.6)	1224 (39.2)	2.52	0.28
(*n* = 3119)	Sometimes	159 (39.6)	966 (35.6)	1125 (36.1)		
	Seldom/never	96 (23.9)	674 (24.8)	770 (24.7)		

ADL, activities of daily living.

**Table 2:** Logistic regression analysis of influencing factors of CMCP service needs.

Variables	OR (95%CI)			
	Model 1	Model 2	Model 3	Model 4
Predisposing variables				
Residence (ref. urban)				
Town	1.94 (1.49, 2.54) ***	1.63 (1.18, 2.24) **	2.04 (1.54, 2.70) ***	1.74 (1.25, 2.43) **
Rural	2.10 (1.49, 2.96) ***	2.07 (1.41, 3.05) ***	2.3 (1.59, 3.31) ***	2.16 (1.44, 3.26) **
Age (ref. 60~69)				
70~79	2.21 (1.48, 3.30) ***	1.65 (1.06, 2.58) *	1.74 (1.13, 2.69) *	1.29 (0.80, 2.08)
≥80	1.31 (0.84, 2.03)	1.09 (0.68, 1.75)	1.04 (0.65, 1.66)	0.87 (0.52, 1.43)
Education (ref. illiterate)				
Primary	0.58 (0.41, 0.80) **	0.77 (0.51, 0.95) *	0.62 (0.44, 0.87) **	0.83 (0.55, 1.26)
Junior high school	0.94 (0.68, 1.29)	1.18 (0.81, 1.73)	0.97 (0.70, 1.35)	1.18 (0.79, 1.74)
Senior high school and above	1.86 (1.11, 2.21) **	1.96 (1.65, 2.40) *	1.81 (1.57, 2.16) *	1.95 (0.64, 3.40)
Enabling variables				
Occupation (ref. farmer)		1.4 (1.03, 1.90)		1.27 (0.93, 1.75)
Number of children (ref. 0)				
1		0.77 (0.17, 3.44)		0.82 (0.18, 3.75)
≥2		1.35 (1.00, 1.84)		1.37 (1.00, 1.87)
Marital status (ref. married)		1.61 (1.14, 2.27) **		1.64 (1.15, 2.34) **
Economic status (ref. expenditures exceeded income)				
Balance		1.71 (1.10, 2.67) *		1.64 (1.03, 2.60) *
Income exceeded expenditures		2.34 (1.92, 3.94) **		2.28 (1.88, 3.88) **
Primary caregiver (ref. none)				
Spouse		0.47 (0.19, 0.81) **		0.32 (0.12, 0.95) **
Children		1.46 (0.43, 4.94)		1.27 (0.37, 4.35)
Others		2.05 (1.60, 4.97) *		1.77 (1.51, 3.12) *
Need variables				
Self-rated health (ref. bad)			1.13 (0.80, 1.58)	1.11 (0.77, 1.61)
ADL (ref. completely independent)				
Relatively independent			2.35 (1.05, 5.26) *	2.13 (1.94, 4.85) *
Disability			3.78 (2.27, 5.30) **	3.69 (2.22, 5.12) **

Note: *** *p* < 0.001; ** *p* < 0.01; * *p* < 0.05.

**Table 3:** The comparison of prediction probabilities and goodness of fit of model I–IV.

	−2LL	−2LL Change Value *	Cox and Snell R2	Cox and Snell R2 Change Value *	Nagelkerke R2	Nagelkerke R2 Change Value *
Model I	1734.464	—	0.050	—	0.094	—
Model II	1792.426	+57.962	0.037	−0.013	0.069	−0.025
Model III	2094.023	+359.559	0.036	−0.014	0.065	−0.029
Model IV	1858.182	+123.718	0.045	−0.005	0.084	−0.010

Note: * Change value is compared with the model I increase or decrease value.

**Table 4:** Odds ratios of anticipated CMCP services (binary logistic regression).

Variables	Personal Care	Medical Care	Psychological Consulting	Health Examination	Social and Recreational Activities	Healthcare Education
Predisposing variables						
Residence (ref. urban)						
Town	1.94(1.49,2.54) **	1.65(1.06,2.58) *	1.63(1.18,2.24) **	2.35(1.05,5.26) *	2.04(1.54,2.70) **	1.74(1.25,2.43) **
Rural	2.10(1.49,2.96) ***	2.30(1.59,3.31) ***	2.07(1.41,3.05) ***	3.78(2.27,5.30) **	2.39(1.28,3.35) **	2.16(1.44,3.26) **
Age (ref. 60~69)						
70~79	1.79(1.42,2.53) *	1.69(1.22,2.13) **	1.19(0.68,1.76)	1.54(1.13,2.50) *	1.14(0.75,1.69)	1.39(0.79,2.28)
≥80	2.31(1.58,3.29) ***	2.17(1.43,3.16) **	1.66(1.16,2.48) *	2.18(1.78,3.68) **	1.64(1.23,2.59) *	1.97(1.42,2.25) **
Education (ref. illiterate)						
Primary	0.59(0.46,0.87) **	0.38(0.13,0.95) **	0.78(0.51,0.94) *	1.28(0.91,1.76)	0.63(0.43,0.89) **	0.81(0.55,1.26)
Junior high school	0.91(0.65,1.24)	1.29(0.37,4.35)	1.19(0.82,1.75)	1.38(1.05,1.87)	0.95(0.74,1.37)	1.19(0.79,1.74)
Senior high school and above	1.84(1.12,2.24) **	1.78(1.52,3.12) *	1.97(1.66,2.41) *	1.65(1.19,2.37) **	1.82(1.58,2.12) **	1.96(1.64,3.40) **
Enabling variables						
Occupation (ref. farmer)	1.25(0.79,1.78)	1.27(0.81,1.79)	1.37(1.23,1.89)	1.31(0.94,1.76)	1.32(0.81,1.79)	1.18(0.82,1.78)
Number of children (ref. 0)						
1	0.61(0.48,3.25)	0.72(0.28,3.65)	0.68(0.27,3.44)	0.59(0.31,0.75) *	0.76(0.38,3.57)	0.81(0.18,3.64)
≥2	1.47(0.33,1.78)	1.27(0.98,1.77)	1.24(0.59,1.74)	1.15(0.61,1.53)	1.46(0.58,1.67)	1.26(0.57,1.76)
Marital status (ref. married)	1.61(1.27,2.34) **	1.54(1.25,2.24) **	1.52(1.24,2.17) **	1.69(1.45,2.31) *	2.24(1.56,2.84) **	1.55(1.15,2.72) **
Economic status (ref. balance),						
expenditures exceeded income	1.74(1.13,2.71) *	1.47(0.36,1.67)	1.61(1.10,2.77) *	1.19(0.78,1.75)	1.74(1.13,2.50) *	1.54(1.03,2.50) *
Income exceeded expenditures	2.38(1.98,3.98) **	1.74(1.35,2.74) **	2.24(1.82,3.94) **	1.75(1.06,2.68) *	2.38(1.58,3.89) **	2.38(1.78,3.87) **
Primary caregiver (ref. none)						
Spouse	0.22(0.32,0.85) **	0.88(0.71,0.95) *	0.57(0.39,0.81) **	0.92(0.38,3.76)	0.52(0.32,0.96) **	0.42(0.22,0.95) **
Children	1.37(0.47,4.25)	1.28(0.71,1.93)	1.56(0.53,4.94)	1.57(0.59,1.86)	1.57(0.47,4.34)	1.37(0.57,4.25)
Others	1.67(1.61,3.22) *	1.76(1.52,2.48) *	2.25(1.70,4.97) *	1.84(1.46,2.35) **	1.67(1.41,3.13) *	1.67(1.31,3.42) *
Need variables						
Self-rated health (ref. bad)	1.31(0.78,1.64)	1.39(0.23,1.90)	1.51(0.87,1.63)	1.37(0.23,1.85)	2.14(1.82,2.94) **	1.41(0.27,1.81)
ADL (ref. completely independent)						
Relatively independent	2.32(1.93,4.75) *	1.45(1.29,2.15) *	2.14(1.84,4.55) *	1.64(1.35,2.23) **	2.25(1.15,5.26) *	2.14(1.84,4.75) *
Disability	3.59(2.32,5.32) **	1.65(1.07,2.56) ***	3.59(2.42,5.41) **	2.56(1.94,3.16) ***	3.68(2.27,5.20) *	3.65(3.16,4.22) ***
Model summary:						
Nagelkerke R^2^	0.21	0.20	0.19	0.19	0.18	0.18
χ^2^ with df = 45 (*p*-value)	126.35(<0.0001)	124.7(<0.0001)	91.28(<0.0001)	101.84(<0.0001)	91.52(0.0004)	83.95(0.0002)
−2Log likelihood	4781.27	1323.18	1659.72	18197.21	1678.63	1715.84

Note: *** *p* < 0.001; ** *p* < 0.01; * *p* < 0.05. The table presents the final results when all sets of variables were entered at once, for the sake of presentational simplification.

## Abbreviations

LTC	Long-term care
CMCP	Combination of medical care and pension
